# Understanding drug-cytokine synergistic toxicity

**DOI:** 10.1038/cddis.2015.321

**Published:** 2015-11-19

**Authors:** F S Wolenski, Y P Dragan

**Affiliations:** 1 Drug Safety Research and Evaluation, Millennium Pharmaceuticals, Inc (a wholly owned subsidiary of Takeda Pharmaceutical Company Limited), 35 Landsdowne Street, Cambridge, MA 02139, USA

The ability to detect and monitor potential toxicities of new clinical candidates is an absolute necessity for any pharmaceutical agent before the clinic. This safety profile is established during pre-clinical development through *in vitro* assays and animal studies. Although toxicities related to the mechanism of action may be readily detected, there is always a concern about the ‘hidden toxicities' that occur when a drug is combined with some other microenvironmental factor in animals or in the clinic. Wolenski *et al.*^1^ described a model of synergistic cytotoxicity between the investigational agent pevonedistat (MLN4924) and the inflammatory cytokine TNF-*α*. The toxicity between pevonedistat and TNF-*α* was confirmed across multiple cultured cell lines, primary hepatocytes, and in rats. These non-clinical findings led to an improved understanding of the potential for clinical toxicities with pevonedistat that can occur in patients with a pre-existing inflammatory state.^2^

Stimulation of the TNF-receptor (TNFR) results in a range of pro-survival outcomes (inflammation, innate immunity, etc.) that are all regulated by the NF-*κ*B transcription factor (Figure 1a).^3^ However, excessive stimulation of the TNF signaling pathway can activate caspase-8, which in turn drives apoptosis.^4^ In cell-based models, TNF-*α* and other pro-inflammatory cytokines were shown to synergize with pharmaceutical compounds to cause drug-induced injuries.^5^ Similarly, *in vivo* models that couple perturbation of Toll-like receptor (TLR) signaling with selected small-molecule pharmaceuticals can trigger toxicity.^6^

Pevonedistat is a small-molecule inhibitor of the NEDD8-activating enzyme (NAE) that has been evaluated in clinical trials for the treatment of cancer.^2^ The role of NAE is to transfer NEDD8, an ubiquitin-like protein, to downstream substrates such as cullin-RING ligases (CRLs).^7^ Proteins ubiquinated by the CRLs are targeted for proteasome-mediated degradation, and impaired turnover of the DNA replication factor CDT1 leads to cell cycle arrest (Figure 1b). In addition, pevonedistat indirectly inhibits NF-*κ*B signaling by preventing the degradation of I*κ*B*α*.^8^ In a phase 1 trial, a subset of patients treated with high doses of pevonedistat experienced adverse events that included elevated hepatic transaminases following the first dose of pevonedistat.^2^ The clinical findings served as the rationale to develop a pre-clinical model of pevonedistat drug-induced toxicity.

The manuscript by Wolenski *et al.*^1^ identified that the combination of pevonedistat and TNF-*α* was cytotoxic both *in vitro* and *in vivo*. A hepatoma cell line became 50-fold more sensitive to TNF-*α* when dosed in combination with pevonedistat. Similarly, although rats tolerated the single agents alone, the combination resulted in liver damage. Additional cell-based characterizations found that pevonedistat and TNF-*α* specifically activated apoptosis, and inclusion of Z-VAD (a pan-caspase inhibitor) switched the mechanism of death to necroptosis (Figure 1c). Cells only tolerated pevonedistat and TNF-*α* when combined with Z-VAD (to block apoptosis) and Necrostatin-1 (to block necroptosis). Trimeric MLKL was also validated as a biomarker of active necroptosis, which is consistent with the literature.^9^

A single protein, caspase-8, was the driver of pevonedistat and TNF-*α* cell death.^1^ This was best illustrated through a knockdown of caspase-8 expression that prevented the pevonedistat and TNF-*α* synergistic cytotoxicity. Qualitatively, caspase-8 activity in the liver was also highest in rats that received the combination treatment. In cells, pevonedistat and TNF-*α* treatment preferentially resulted in the accumulation of the p10 protease subunit of caspase-8. This novel finding was interpreted as evidence for increased caspase processing/activation and not due to impaired protein degradation.

There remains a gap in the understanding of how pevonedistat sensitizes cells to cytotoxic TNF signaling (illustrated by a ‘?' in Figure 1c). Specifically, one might determine which proteins in TNFR-to-caspase-8 pathway are affected by pevonedistat. Wolenski *et al.* investigated a known link between the CRL member cullin-3 and caspase-8 ubiquitination.^10, 11^ However, there was no evidence that caspase-8 was ubiquitinated in the pevonedistat model.^1^ Knockdown of cullin-3 expression, which was hypothesized to make cells sensitive to TNF-*α* by mimicking pevonedistat, instead limited cell death caused by TNF-*α*. Thus, the exact mechanism of the toxicity requires additional work to fully characterize.

The clinical findings with pevonedistat necessitated an investigative effort to establish a pre-clinical model. Two key lessons were learned from this effort. First, a pre-clinical screening strategy should be employed to identify potential synergistic cytotoxicities between compounds and pro-inflammatory agents. Second, attention needs to given to determine whether regulated necrosis pathways, such as necroptosis, can contribute to drug-induced toxicities. There are multiple types of regulated necrosis, all of which cause the dysregulation of the redox metabolome.^12^ Although pevonedistat only activated necroptosis in cultured cells under specific conditions, the results from Wolenski *et al.* suggest that compounds that affect the TNFR pathway may have similar effects.

From a broader perspective, the dysregulation of cell death is at the center of essentially every form of liver disease.^13^ It is relevant to the understanding of liver toxicity to determine what activates these pathways, how these triggers and pathways influence liver toxicity, and to develop a cell-health assessment strategy for pre-clinical testing. This might be accomplished through a high-content imaging assessment such as proposed by Xu;^14^ an *in vitro* screen such as described by Cosgrove;^5^ and *in vivo* models such as those developed by Roth.^15^ The objectives of all of these approaches is to identify and characterize the mechanisms that drive toxicity, and to define a set of comprehensive assays that improve early safety screening. Validation of these assays with existing compounds, such as pevonedistat, could lead to a better understanding of potential toxicities before compounds enter the clinic.

## Figures and Tables

**Figure 1:**
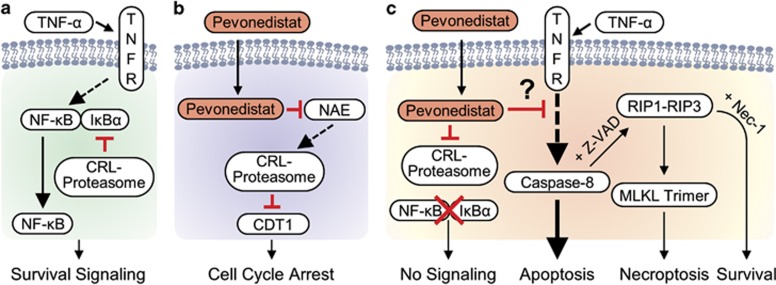
Pevonedistat synergizes with TNF-*α* to induce cell death. (**a**) Stimulation of the TNFR activates NF-*κ*B pro-survival signaling. NF-*κ*B is regulated by the degradation of its inhibitor I*κ*B*α* through the CRL-proteasome pathway. (**b**) Pevonedistat is an inhibitor of NEDD8-activating enzyme (NAE). Inhibition of NAE and thus the CRL-proteasome prevents the degradation of many proteins, such as CDT1 that in turn leads to DNA re-replication and cell cycle arrest. (**c**) The combination of pevonedistat and TNF-*α* kills cells through apoptosis. A pan-caspase inhibitor (Z-VAD) prevents apoptosis but cells then die by necroptosis through a RIP1-RIP3-MLKL mechanism. Combined with Z-VAD, the RIP1 inhibitor Necrostatin-1 (Nec-1) prevents cell death. Pevonedistat blocks NF-*κ*B signaling and has a putative effect on an unknown aspect of pathway that links the TNFR to caspase-8. Solid lines indicate a direct link, whereas dashed lines represent multiple intermediate steps
